# A rare case of intranodal hemorrhagic spindle cell tumor with amianthoid fibers presenting as a suspicious lump in the groin

**DOI:** 10.1016/j.ijscr.2019.09.042

**Published:** 2019-10-07

**Authors:** Esra Nsour, Ali Al Khader

**Affiliations:** a Department of Pathology, Al Hussein Salt Hospital, Ministry of Health, Al-Salt, Jordan; b Full-time Lecturer of Pathology, Faculty of Medicine, Al-Balqa Applied University, Al-Salt, Jordan

**Keywords:** Lymph node, Myofibroblastoma, Spindle cell

## Abstract

**Introduction:**

Intranodal hemorrhagic spindle cell tumor with amianthoid fibers is extremely rare.

**Presentation of the case:**

Herein, we present the case of a 58-year-old man with a 2-year history of painless left inguinal swelling. On physical examination, the mass was large, fixed, hard, and non-tender, suggesting a lymph node pathology. The mass had increased slightly in size after a short course of antibiotic treatment. The surgeon decided to perform an excisional biopsy of the left inguinal lymph node. The lesion was intraoperatively hard and suspicious for malignancy. The gross examination revealed a relatively large well-circumscribed mass with a whitish, hard, cut surface. The histomorphological and immunohistochemical findings were consistent with intranodal hemorrhagic spindle cell tumor with amianthoid fibers. There was no evidence of recurrence at 4 years post surgery.

**Discussion:**

The present case adds to the very limited data available on this rare entity.

**Conclusion:**

Intranodal hemorrhagic spindle cell tumor with amianthoid fibers can be a diagnostic pitfall both clinically and histopathologically. It can reach large sizes and be clinically and grossly suspicious for lymphoma. Clinicians must be made aware that such an entity exists.

## Highlights

• Hemorrhagic spindle cell tumor with amianthoid fibers is extremely rare.
• The present case adds to the very limited data available on this entity.
• The tumor in the present case reached a relatively large size and raised clinical suspicion for lymphoma.
• This tumor is a mimicker for many benign and malignant conditions.
• This tumor must be considered to avoid the misinterpretation as primary malignant spindle cell lesion or metastasis.

## 1 Introduction

Intranodal hemorrhagic spindle cell tumor with amianthoid fibers, also known as intranodal palisaded myofibroblastoma, is a benign mesenchymal tumor that is extremely rare, with only a small number of reported cases in the English literature [1,2]. It arises from the lymph node tissue and shows dual mesenchymal differentiation toward smooth muscle cells and myofibroblasts [3]. Although the tumor can occur at any age, it usually manifests in adults in their 40 s and 50 s [4]. Because of its striking similarity to peripheral nerve sheath and smooth muscle tumors, the tumor had been variably diagnosed in the past as an intranodal schwannoma or a leiomyoma or their malignant counterparts [5]. Grossly, these tumors show gray-white cut surfaces with interspersed regions of focal hemorrhage. A compressed rim of residual nodal tissue can often be identified [4]. Microscopically, the tumor cells are bland looking, with minimal to absent mitotic activity. Although hemosiderin and extravasated red blood cells are characteristic of this tumor, the presence of thick collagen bands in different planes, known as amianthoid fibers, is the most distinctive microscopic feature. In line with SCARE criteria [6], we present the case of a 58-year-old man with a 2-year history of left inguinal swelling. The histomorphological and immunohistochemical findings were consistent with intranodal hemorrhagic spindle cell tumor with amianthoid fibers.

## 2 Report of the case

**Fig. 1:**
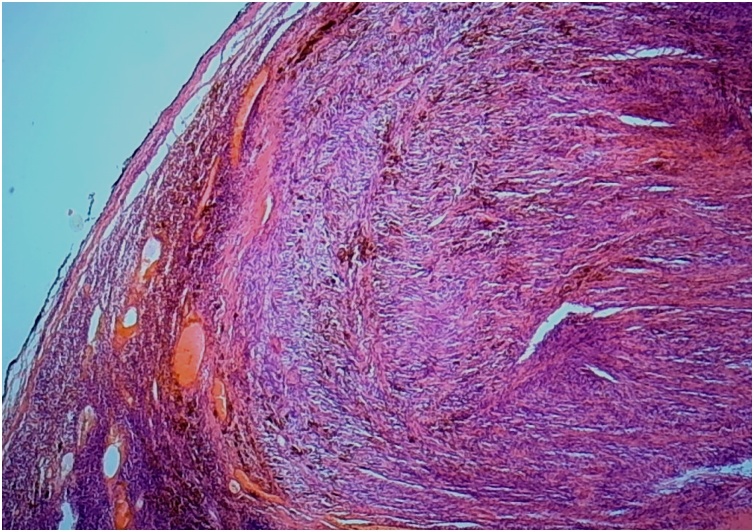
An intranodal spindle cell lesion is seen at low power (HE staining, ×40).

**Fig. 2:**
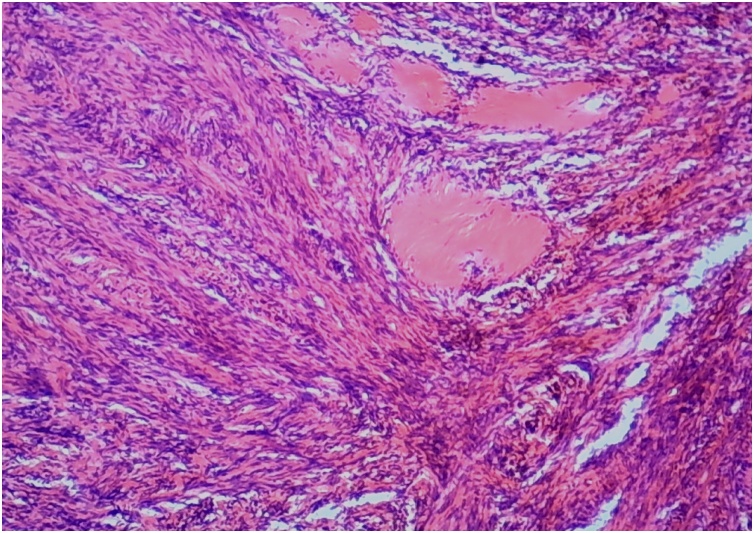
The tumor cells are bland looking and are focally palisaded around the amianthoid fibers (HE staining, ×100).

**Fig. 3:**
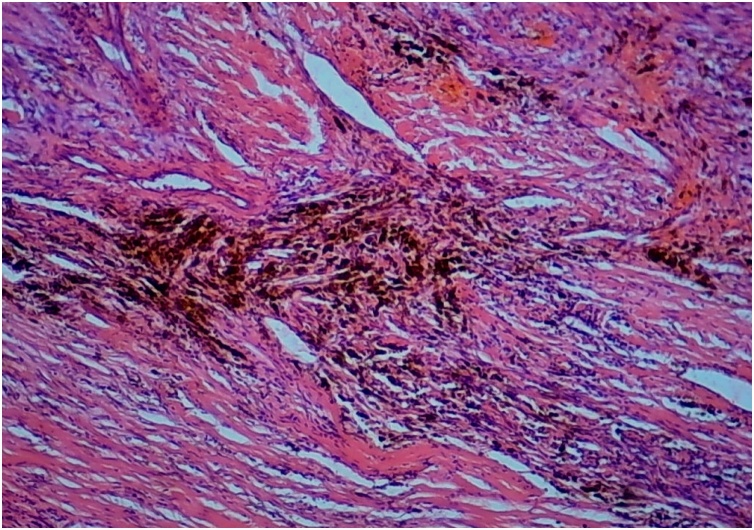
Foci of hemosiderin deposition are characteristic (HE staining, ×100).

**Fig. 4:**
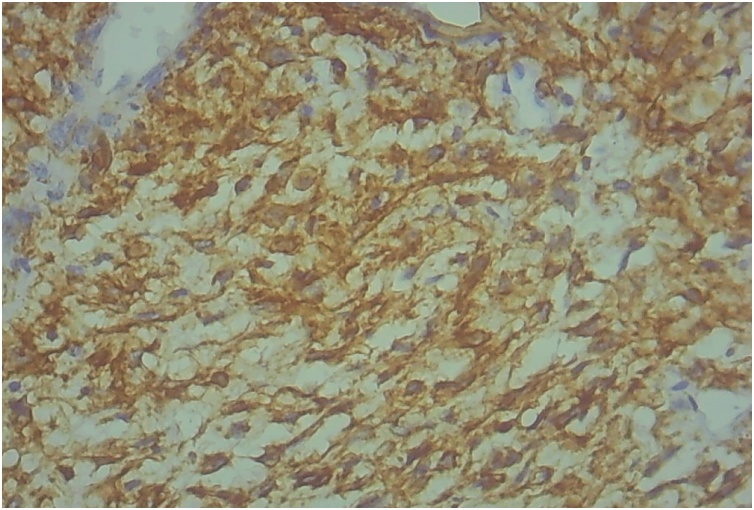
The tumor cells are immunohistochemically positive for smooth muscle actin (×400).

This 58-year-old man, previously otherwise healthy, presented to the outpatient surgical clinic with a 2-year history of left inguinal swelling. On physical examination, a large, hard, fixed, non-tender lump in the left groin was found, suggesting inguinal lymphadenopathy. The mass had increased slightly in size after a short course of antibiotic treatment. The surgeon decided to perform an excisional biopsy of the left inguinal lymph node. The lesion was intraoperatively suspicious because it was relatively large and hard. The procedure was uneventful. Gross examination revealed a well-circumscribed tumor measuring approximately 4.5 cm in maximum dimension and pushing the residual lymph node tissue at the periphery. A whitish, hard, cut surface with small hemorrhagic foci was seen. Microscopically, an intranodal tumor composed of fascicles of bland-looking spindle cells was identified (Fig. 1). Nuclear palisading foci surrounding thick eosinophilic bundles were present (Fig. 2), in addition to multifocal red blood cell extravasation and hemosiderin deposition (Fig. 3). Mitotic activity was absent. The surrounding lymph node tissue showed reactive changes, with foci of hemosiderin deposition as well. The tumor cells were immunohistochemically positive for smooth muscle actin (Fig. 4), and negative for pancytokeratin, CD31, CD34, S100, MART-1, and desmin. The Ki67 proliferative index was low. Masson trichrome staining highlighted the collagen bundles previously described. The case was signed out as intranodal hemorrhagic spindle cell tumor with amianthoid fibers (intranodal palisaded myofibroblastoma). There was no evidence of recurrence at 4 years post surgery.

## 3 Discussion

Reported by Suster and Rosai as hemorrhagic spindle cell tumor with amianthoid fibers [7] and by Weiss et al. as palisaded myofibroblastoma [5], the rarity of this tumor and the overlap of its microscopic appearance with that of many benign and malignant conditions make it a significant diagnostic pitfall that must be dealt with carefully. The present case was of a middle-aged male. Several reports have documented a male-to-female ratio of approximately 2:1 for this tumor [4]. Although the groin is the most commonly reported site, other locations have been reported in a few cases [2]. The large tumor size in the present case had initially raised clinical suspicion of a lymphoproliferative process. Moreover, on physical examination, the lump was hard and fixed. This case demonstrated that palisaded myofibroblastoma can mimic a lymphoma clinically. Moreover, the tumor increased in size after a short course of antibiotic treatment, which further made the diagnosis challenging.

Microscopically, the nuclear palisading noticed in the present case closely mimicked the Verocay bodies seen in schwannomas. However, because the tumor was immunohistochemically negative for S100, an intranodal schwannoma was excluded. In fact, because of the striking resemblance to schwannomas, early authors had regarded this tumor as a schwannoma of the lymph node [8]. The cellularity and extravasation of red blood cells simulated a Kaposi sarcoma, necessitating a test for CD34, which is positive in Kaposi sarcoma. In addition, Kaposi sarcoma is negative for smooth muscle actin and shows increased mitotic activity [4,9]. The immunohistochemical negativity for desmin excluded a leiomyoma. Because a metastatic melanoma in the lymph node commonly exhibits a spindle cell morphology, immunohistochemical staining for S100 and MART-1, which are positive in melanomas, was fundamental to exclude such feared scenario [10]. Moreover, the immunohistochemical negativity for pancytokeratin excluded a metastatic spindle cell carcinoma.

The tumor in the present case showed a benign behavior, with no evidence of recurrence at 4 years post surgery. This was comparable to other reports that revealed a recurrence rate of less than 10%. Moreover, there are no reported cases of malignant transformation or distant metastasis of this tumor [4].

## 4 Conclusion

Intranodal hemorrhagic spindle cell tumor with amianthoid fibers is an extremely rare entity that represents a diagnostic pitfall both clinically and histopathologically. Moreover, the tumor can reach large sizes and be clinically and grossly suspicious for lymphoma. Clinicians must be made aware that such a disease entity exists.

## Sources of funding

This research did not receive any specific grant from funding agencies in the public, commercial, or not-for-profit sectors.

## Ethical approval

This case report is exempt from ethical approval in our institution.

## Consent

Written informed consent was obtained from the patient for publication of this case report and accompanying images.

## Author contribution

Esra Nsour: Conceptualization, data curation, investigation, methodology, supervision, validation, visualization, Writing-original draft and Writing-review and editing.

Ali Al Khader: Investigation, methodology, validation, Writing-original draft and Writing-review and editing.

## Registration of research studies

NA.

## Guarantor

Esra Nsour.

## Provenance and peer review

Not commissioned, externally peer-reviewed.

## Declaration of Competing Interest

The authors declare that they have no conflict of interest.
